# Alumina-based Coating for Coke Reduction in Steam Crackers

**DOI:** 10.3390/ma13092025

**Published:** 2020-04-26

**Authors:** Stamatis A. Sarris, Steffen H. Symoens, Natalia Olahova, Marie-Françoise Reyniers, Guy B. Marin, Kevin M. Van Geem

**Affiliations:** Department of Materials, Textiles and Chemical Engineering, University of Gent, Technologiepark 914, 9052 Gent, Belgium; stamatisasarris@gmail.com (S.A.S.); steffen.symoens@ugent.be (S.H.S.); natalia.olahova@kubota.com (N.O.); MarieFrancoise.Reyniers@UGent.be (M.-F.R.); guy.marin@ugent.be (G.B.M.)

**Keywords:** CoatAlloy™, passivating coating, steam cracking, thermal cracking, ethane, coke formation, Ni-Cr alloy, aging, jet stirred reactor

## Abstract

Alumina-based coatings have been claimed as being an advantageous modification in industrial ethylene furnaces. In this work, on-line experimentally measured coking rates of a commercial coating (CoatAlloy™) have pointed out its superiority compared to an uncoated reference material in an electrobalance set-up. Additionally, the effects of presulfiding with 500 ppmw DMDS per H_2_O, continuous addition of 41 ppmw S per HC of DMDS, and a combination thereof were evaluated during ethane steam cracking under industrially relevant conditions (T_gasphase_ = 1173 K, P_tot_ = 0.1 MPa, X_C2H6_ = 70%, dilution δ = 0.33 kg_H2O_/kg_HC_). The examined samples were further evaluated using online thermogravimetry, scanning electron microscopy and energy diffractive X-ray for surface and cross-section analysis together with X-ray photoelectron spectroscopy and wavelength-dispersive X-ray spectroscopy for surface analysis. The passivating coating illustrated a better performance than the reference Ni-Cr Fe-base alloy after application of an improved pretreatment, followed by piddling changes on the product distribution. Presulfiding of the coating affected negatively the observed coking rates in comparison with the reference alloy, so alternative presulfiding and sulfur addition strategies are recommended when using this barrier coating.

## 1. Introduction

Industrial steam cracker economics suffer from a notorious enemy: carbon formation. On the inner wall of the reactor tubes, carbon filaments are catalytically growing starting from small whiskers leading to a porous layer of interwoven filaments. Heavy hydrocarbons together with dehydrogenated complex carbon chains grow or are deposited on these filaments and form a layer that is well-known as coke [1]. During coke formation, the reactor cross-sectional area is reduced, increasing the pressure drop over the length of the steam cracker [2]. Consequently, the ethylene selectivity decreases [3,4], while the highly insulating coke layer blocks the heat transfer from the burners to the reactive gas. In order to keep the process productivity stable over the run length, i.e., counterbalance the additional heat resistance, the energy load to the process increases, leading to increased fuel consumption and reactor tube metal temperature. Eventually, the reactor tube metal temperature or the pressure drop exceeds a maximal threshold and a procedure in which typically a steam/air mixture is fed to remove the coke is used [5,6,7,8]. Due to this “decoking” procedure, the annual production capacity is limited and the operating costs rise.

To restrain this financial loss, numerous “anti-coking” technologies have been introduced and suggested in the industrial arena of steam cracking, frequently categorized in various groups: three-dimensional (3D) reactor technologies [9,10,11,12,13,14,15], feed additives [16,17,18,19,20,21,22,23,24] and surface technologies [25,26,27,28,29,30,31]. Surface technologies [18], and in particular coatings, have been claimed to completely solve the coking problem. Barrier coatings aim at the inner wall passivation against coke formation by covering the active sites responsible for catalytic coke formation. However, the non-catalytic coke, formed through a free-radical mechanism—often termed pyrolytic coke—is not prevented. As the pyrolytic cokes grows first in between the catalytically formed whiskers or on top of the whiskers it can be expected that by blocking the catalytic active sites also the pyrolytic coke will be affected. In addition, catalytic coatings aim, not only to eliminate catalytic coke formation by covering the active sites, but also to provide catalytic sites for converting pyrolytic coke to carbon oxides and hydrogen through gasification reaction with steam. The main drawback of the latter is a potential high formation rate of carbon oxides and the longevity of the coating activity.

To date, several barrier coatings have been introduced for steam cracking reactors [25,32,33,34,35]. The performance of an Al/Si barrier coating that is applied in a two-step chemical vapor deposition process and suppress the total amount of coke up to 90% for ethane cracking and up to 80% for naphtha cracking using as reference material HP 40 was commercialized as AlcroPlex^®^ and evaluated by Zychlinski et al. [32]. Ganser et al. [33] tested AlcroPlex^®^ in an industrial ethane cracker, observing double run lengths followed by lower CO formation, but also decreased in half decoking time. As additional benefit no tube carburization was observed, while the coating seemed essentially unchanged after a year. Nova Chemicals (PA, USA) and Kubota (Orillia, Ontario, Canada) have developed ANK 400, an inert micron-sized manganese chromium oxide spinel, to diminish both catalytic and pyrolytic coke [34,35], evaluated in two coated ethane cracking furnaces. The results showed more than a factor 10 run length increase in the first run, while subsequent runs indicated an increased duration of around 175 days.

Another technology to reduce the coking tendency of alloys is the application of the catalytic coatings on top of the alloy. The aim of these catalytic coatings is the gasification of the coke deposited on the inner surface of the tube leading—in theory—to an absolute minimization of coke deposition. The Catalyzed-Assisted Manufacture of Olefins—“CAMOL”—coating was developed by Quantiam Technologies (Edmonton, Alberta, Canada) and Nova Chemicals and it has been commercialized by BASF Qtech since 2011 [28,36,37,38]. SK-Corporation (Seoul, South Korea) developed a method of online coating the reactor inner walls with a catalytic film, called PY-COAT [39]. Application in a Millisecond naphtha cracking furnace more than doubled the run length. A novel catalytic coating by GE (Schenectady, NY, USA), called ‘YieldUp’, was evaluated under ethane steam cracking conditions both in a pilot plant and a laboratory scale reactor by Schietekat et al. [29]. The run length increased by a factor 6 with an increase in the formation of carbon oxides. Recently, Mahmoudi et al. [40] have investigated the effect of a CeO_2_-promoted coating, aiming at gasification of the coke layer, that overall inhibited coke formation up to a factor 2. However, no results were reported regarding the formation of carbon oxides.

Westaim Surface Engineered Products (Edmonton, Canada) developed another barrier coating, called CoatAlloy™ [41,42,43], consisting of an engineered surface, an enrichment pool and diffusion barriers coated on the untreated alloy. In the original patent [44], the intermediary diffusion barrier is an alumina-containing coating deposited directly onto the untreated alloy prior to deposition of the enrichment pool. The enrichment pool is a MCrAlX material in which M can alter among Ni, Co, Fe or an alloy and X is Y, Hf, Zr, La or a combination thereof. This enrichment pool and the untreated alloy are heat-treated to metallurgical bond the coating and to form a multiphase microstructure. The overlay coating is then aluminized by depositing a layer of alumina and oxidizing the resulting coating to form an alumina surface layer. Between 1995 and 2001 several improvements have been made to the technology resulting in an increased operating limit from 1293 K (Original CoatAlloy^TM^) to 1333 K (CoatAlloy^TM^–1060) and 1373 K (CoatAlloy^TM^–1100) [43]. By 2001, CoatAlloy^TM^ coatings were installed in 25 furnaces globally and typically resulted in a decrease in coking rate by about 90% with no reported effect on carbon oxides formation [43]. Recently, Olahová et al. [45], evaluated the performance of CoatAlloy™ at a pilot plant, by measuring the total amount of coke and providing CO and CO_2_ yields. In all coating technologies no exact details are provided on decoking, pretreatment or cracking conditions, nor a one-to-one comparison is done, therefore the real impact of one particular element to coke formation is difficult to assess.

In this work, a passivating alumina coating called CoatAlloy™ is tested for its anti-coking abilities by comparing its coking behavior with that of a reference CrNi alloy, representing the current state of the art. Essential with all coatings applied on high temperature alloys is if they actually keep their initial performance and therefore multiple cracking/decoking cycles are carried out on a jet stirred reactor (JSR) set-up under industrially relevant conditions in combination with different pretreatment methods. This allows for the first time to quantitatively assess the performance of this alumina coating and understand—supported by a variety of surface analysis (SEM and EDX, XPS and WDS) results—how the coating interacts with coke and how the coating composition evolves over time. All these give the maximum insight on the occurring phenomena, leading to the selection of the optimal conditions for minimizing coking with the presented passivating technology.

## 2. Experimental Section

### 2.1. Electrobalance Set-up

**Figure 1:**
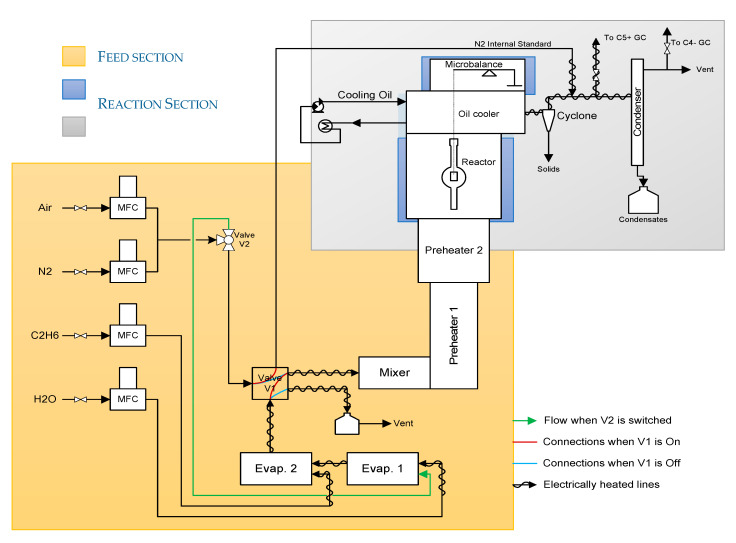
Schematic representation of the JSR set-up.

The coking experiments were performed in a jet stirred reactor set-up equipped with an electro- balance [7,27]. A schematic representation of the setup can be found in Figure 1.

The JSR is designed to carry out thermogravimetric analyses on different reactor materials. Coke formed during the experiments can be measured as a function of time under industrial relevant conditions. The unit is capable of evaluating the effect of different pretreatment conditions (e.g., application of additives, temperature and duration of the pretreatment), as well as varying cracking conditions (e.g., use of the feed additives, steam dilution and cracking temperature) on coke formation. The product distribution together with the surface composition and morphology can be measured for further investigation. During an experiment, the coke deposited on the metal sample is accurately monitored online and processed to obtain coking rates. The reactor effluent is online analyzed via gas chromatography to evaluate the cracking severity. The surface morphology and chemical composition of the samples are analyzed by means of SEM and EDX, using a JEOL analyzer, type JSM-7600F (University of Gent, Zwijnaarde, Belgium), with a Schottky field emission gun as an electron source. The combination of the mentioned set-up together with the experimental procedure gives valuable information on the coke formation phenomenon occurring during steam cracking of hydrocarbons and leads to a rather global explanation of the influence of the studied material and conditions.

The experimental unit is described in detail in previous work [7,30,31]. In the feed section, the hydrocarbon feedstock and water (or H_2_O with DMDS) are fed, evaporated, mixed and preheated before the mixture is sent to the reactor section. There, the steam cracking process is occurring, which leads as side reaction to coke formation on the sample. The reactor effluent is quenched downstream using an oil cooler and a fraction of the effluent is withdrawn for online *C*_4−_ and *C*_5+_ gas chromatographic analysis, while the rest is sent directly to the vent and liquid waste. In this way all hydrocarbons up to C_10_ can be measured, next to H_2_, CO and CO_2_ [46].

### 2.2. Samples: Materials and Preparation

**Figure 2:**
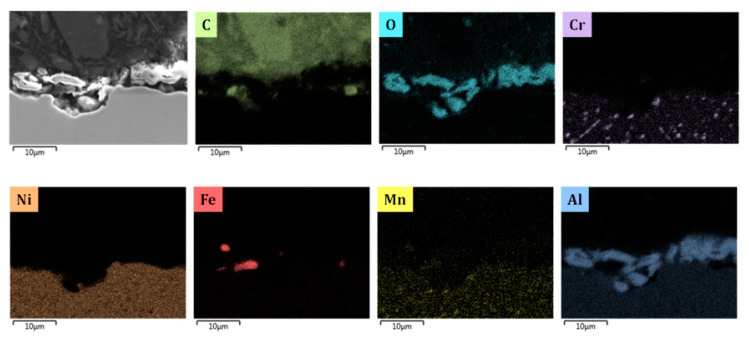
Cross-sectional elemental mappings of a fresh CoatAlloy™ sample. Magnification: 3000 ×, Accelerating Voltage: 15 kV.

The metal samples are made out of 25/35 Cr/Ni obtained from Manoir Global Solutions (Pitres, France) and are cut as close as possible to the internal surface of industrial tubes using wire-cut electrical discharge machining (EDM) (AGIE Excellence 2F eCut, wire-cut EDM, University of Gent, Zwijnaarde, Belgium) with an accuracy of 1 μm in 10 × 8 × 1 mm^3^ dimensions (total surface area S = 1.96 × 10^−4^ m^2^). The composition of the reference alloy is given in Table 1. The composition of CoatAlloy™ is schematically illustrated in Figure S1. CoatAlloy™ is a multilayered coating with a diffusion barrier which serves to isolate the coating from the reference alloy. The top surface is an aluminum-containing layer which gives the material its anticoking performance. The coating is applied on the inner surface of the cracking coil which is in this case a sample of 25/35 Cr/Ni. The sample is heat-treated just after the deposition step to activate it and consists based on the SEM_EDX results shown in Figure 2 of Al, O on top of the reference 25/35 Cr/Ni.

### 2.3. Experimental Procedures and Conditions

Each experiment consists of eight cracking cycles followed by their decoking procedure. Before the first cracking cycle, the sample surface is properly oxidized using a mixture of steam, air and/or nitrogen, as described in the Introduction [5,6,7,8]. During a cracking cycle the reactant gas is fed to the reactor and cracking takes place. Throughout cracking, coke is depositing on the metal sample. Cracking duration is usually 6 h. After cracking, a decoking procedure, similar to the applied pretreatment is also performed to regenerate the oxides formed on the sample’s surface and to burn off the deposited coke [7]. The cyclic aging experiment conducted in the JSR is known to be representative of the industrial coking behavior of an alloy [48]. It consists of three 6 h cracking and decoking cycles, followed by four short cracking cycles of 1 h and a last cracking cycle of 6 h to evaluate the effect of cyclic aging on each applied set of conditions, so in total eight cycles of coking, each followed by decoking. An overview of the experimental sequence followed in the aging experiments together with detailed information on the conditions for each process step is given in Table 2. 

**Figure 3:**
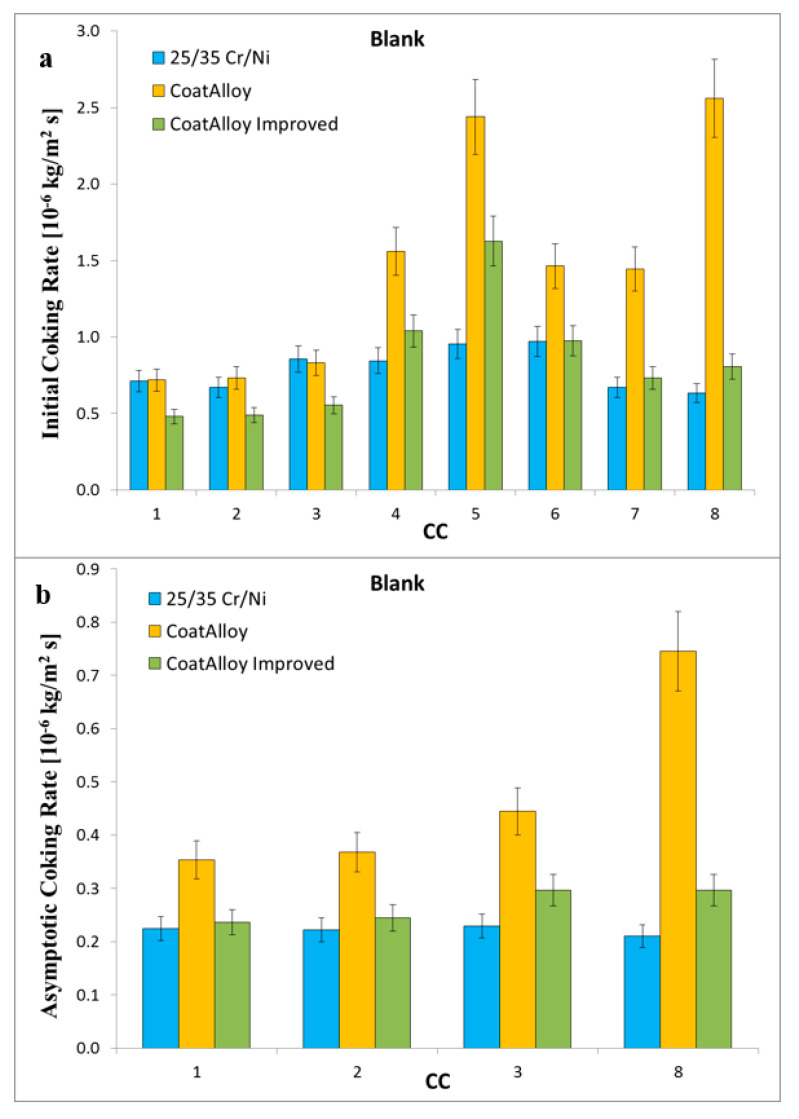
Initial (**a**) and asymptotic (**b**) coking rates for the Blank runs for the reference (blue), CoatAlloy™ (yellow), and CoatAlloy™ with the Steam/Air pretreatment (green).

The pretreatment used for the reference is the standard one for Fe-Ni-Cr alloys and published in previous work [7]. In case of presulfiding of the reactor, the same sequence of pretreatment and decoking is followed before presulfiding. Presulfiding is practically an additional last step applied after the pretreatment or the decoking. The samples are presulfided at 1100 K with a diluted mixture of DMDS (500 ppmw DMDS per H_2_O). Cracking of ethane was performed at similar conditions with previous work [7,29] (see Table 2). In the case of the alumina-based coating a slightly different pretreatment—or decoking after cracking—step is followed. This is because it has been observed experimentally that nitrogen has a negative influence on the formation and preservation of the Al-oxides [49,50,51], as it could penetrate through the oxide and react with Cr by precipitation of nitrides. The potential formation of carbon nitrides can possibly generate a deformation of the coating due to mechanical stresses during cooling down and heating up. Also in this work—see comparison in Figure 3—it is observed that N_2_ increases the coking rate for the alumina-based coatings. Hence, only steam and air was fed to the reactor for 45 min for all the CoatAlloy™ experiments, instead of the typical procedure that is described above for the Fe-Ni-Cr alloys. In this work the pretreatment used for the alumina-based coating is a steam/air treatment for the coating—in comparison with the classical for Fe-Ni-Cr alloys. During cooling down an inert atmosphere of He is fed to the reactor for all the materials. To summarize, four different cases are evaluated for which coking rates, effluent analysis and surface analyses were obtained:

- A blank experiment (referred to as ‘Blank’): only ethane and steam are fed during the experiment
- A presulfiding experiment (referred to as ‘PreS’): a presulfiding step is applied before cracking, but for the rest identical to ‘Blank’
- A continuous addition experiment (referred to as ‘CA’): ethane and steam with 41 ppmw S per HC are fed to the reactor continuously during cracking.
- A continuous addition combined with CA, (referred to as ‘CA + PreS’): a presulfiding step is applied, during cracking ethane and steam with 41 ppmw S per HC are fed to the reactor continuously.

### 2.4. Coking Rate Determination

Similar to previous work [7,26,27], during the cracking experiment, relevant experimental data is logged, such as temperatures, mass flows and the mass of the deposited coke. The coke deposition on each sample is measured over time by continuously weighing the mass of the sample. This allows the determination of the total amount of coke after every cracking cycle, as well as the calculation of the initial catalytic coking rate and the asymptotic pyrolytic coking rate. The calculated coking rate, from the balance signal between 15 and 60 min, is considered as representative for the catalytic behavior of the material, while the rate calculated from the 5^th^ to 6^th^ hour is referring to the long-term behavior of the material, i.e., the pyrolytic coking behavior, occurring when the complete surface of the sample is covered by coke. In the remainder of this work, the former is referred as initial coking rate while the latter as asymptotic. A more detailed description of the coking curve processing is included in the supporting information.

### 2.5. Surface Characterization

Scanning Electron Microscopy (SEM) and Energy Dispersive X-ray spectroscopy (EDX) are used to obtain information regarding the surface morphology and elemental composition of the samples. The top surface analysis gives a qualitative idea of the coke growth together with elemental quantitative data of the surface, performed in 10 kV and 20 kV. The uniformity of the oxides formed on the surface is evaluated with the cross section elemental mappings.

Additionally, in this work, wavelength-dispersive spectroscopy (WDS) are conducted at a WDX-apparatus from Oxford Instrument (University of Gent, Belgium) and X-ray photoelectron spectroscopy (XPS) (S-probe Monochromatized XPS Spectrometer, Surface Science Instruments, University of Gent, Belgium) are used for some of the samples to gain additional insight on the influence of nitrogen and sulfur on the metal surface. For WDS, the detection of sulfur is performed using a pentaerythritol (PET) crystal, which allows detection of sulfur levels as low as 0.1 wt%. In Table 3, the location of the XPS characteristic peaks of the most important elements for the studied materials is given.

## 3. Results and Discussion

### 3.1. Products and Coke Formation

Averaged yields of the main products, together with initial and asymptotic coking rates for the performed cracking experiments on the reference and CoatAlloy for the two pretreatments and the sulfur addition during the cyclic aging procedure are summarized in Table 4.

The influence for every set of conditions was investigated while performing a cyclic aging of the samples. It should be highlighted that the coking results after aging are the most representative of the coking behavior of an industrial cracker operating under similar conditions. Overall, the gas phase composition is not affected for the different conditions apart from the formation of carbon oxides.

The addition of sulfur during cracking or as pretreatment led to carbon oxides decrease. Significant differences were noted between the reference material and the passivating formulation under the studied conditions—except for the blank runs. During the blank experiments, almost two times more CO was measured for the passivating coating compared to the reference. Application of an additional presulfiding step before every cracking cycle led to a 20% decrement of carbon oxides formation for both the reference and the coated sample.

Comparing the blank run with the one that sulfur was continuously added, the reduction of CO and CO_2_ formation was more pronounced; five and two times the mitigation of CO and CO_2_, respectively, was observed for the reference alloy, while for CoatAlloy™ this was even more pronounced, with nine and two times lower amounts, respectively. After combining presulfiding and continuous sulfur addition, no substantial difference was noticed in comparison with the continuous addition only effect when it is compared to the blank runs, so practically presulfiding has no added value for the mitigation of carbon oxides when continuous addition is already applied.

Figure 3 shows the initial and asymptotic coking rate for the blank run conditions: CoatAlloy™ cokes two to five times more than the reference, when the same treatment was used. In the case that the treatment for CoatAlloy™ did not include N_2_ and only steam/air mixture was applied, the asymptotic coking rate observed was comparable with the one of the reference material, it even decreased by a factor of three in comparison with the standard treatment.

**Figure 4:**
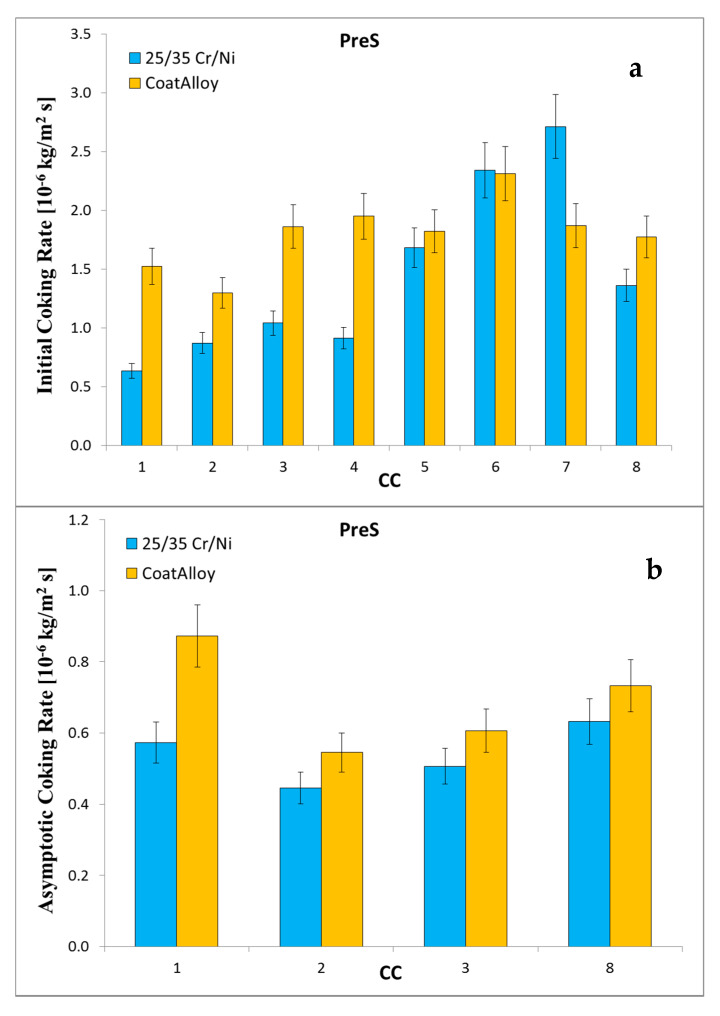
Initial (**a**) and asymptotic (**b**) coking rates for the Presulfiding runs for the reference (blue) and CoatAlloy (yellow) sample.

Adding a presulfiding step to the blank experiments—denoted as PreS experiments—results in an increase with an average increase a factor of two of the asymptotic coking rate compared to the blank conditions for both samples, as can be observed in Figure 4. More importantly after cyclic aging the asymptotic coking rate of CoatAlloy™ decreases by 15%, while the asymptotic coking rate for the reference remains rather stable. Overall, it can be concluded that under these presulfiding conditions the two samples perform similarly.

**Figure 5:**
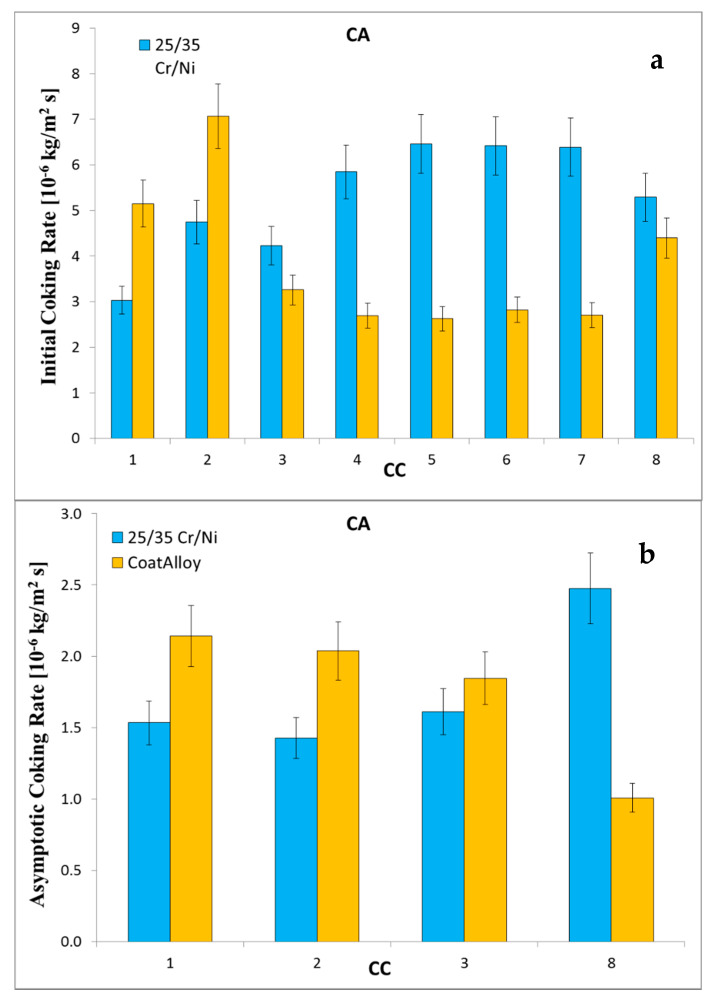
Initial (**a**) and asymptotic (**b**) coking rates of CA experiments.

When DMDS is continuously added, initially the reference performs about two times better than CoatAlloy™, as can be observed in Figure 5. However, by applying a cyclic aging, the performance of the barrier coating improves significantly, while the reference material remains rather stable. In that way, the application of the coating results in 10 and 50% lower coking rates for the initial and asymptotic, respectively.

The general reason behind the better anti-coking performance after the cyclic aging can be due to the formation of a more stable α-Al_2_O_3_ oxidized phase on the surface of the coating after several of coking-decoking cycles or after long exposure to steam treatment.

**Figure 6:**
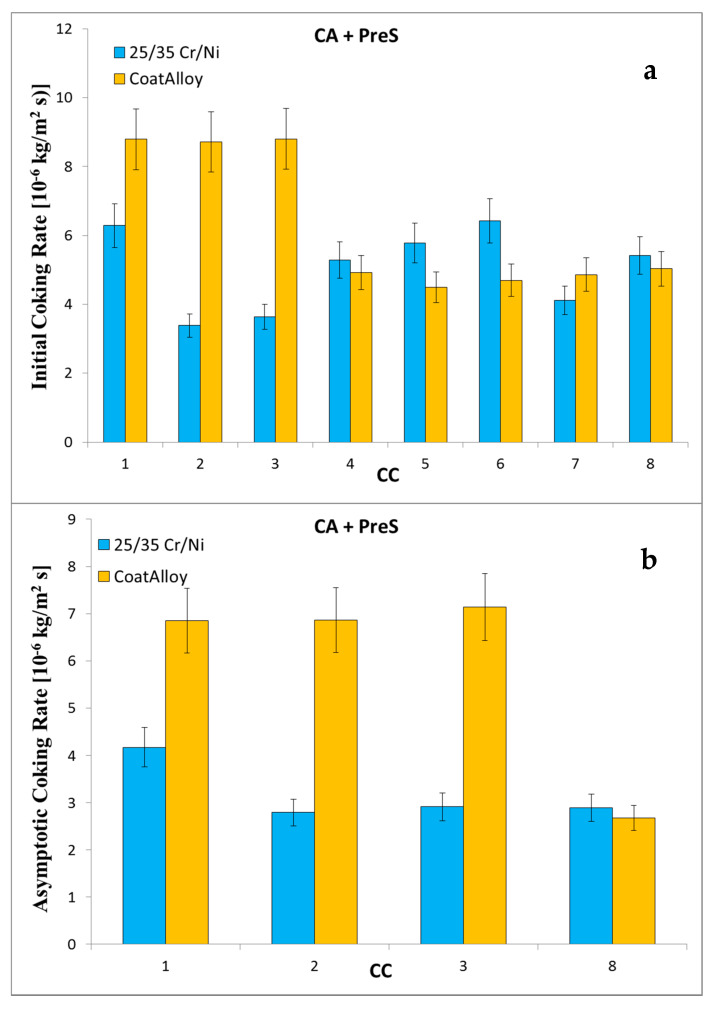
Initial (**a**) and asymptotic (**b**) coking rates of PreS + CA experiments.

If presulfiding is implemented to the continuous addition conditions—the case of the PreS + CA experiments—then, during the first cycles, the reference performs significantly better than CoatAlloy™, as illustrated in Figure 6. Nevertheless, again the coating seems to perform a lot better after cyclic aging giving the impression that with one additional cycle would outrun the reference material in anti-coking performance. It is in general suggested that presulfiding should be avoided when applying the passivating coating on the coil.

### 3.2. Surface Analysis

All samples were examined for both top surface and cross section analysis. The idea is to observe structural and compositional differences by the top surface analysis. Using the latter, clear conclusions can be made. The cross-section elemental analysis evaluates the homogeneity, thickness and qualitative composition of the oxide layers formed on the surface. In an attempt to obtain insight on the effect of N_2_ and sulfur on the surface of the coating, additional analysis with X-ray photoelectron spectroscopy (XPS) and wavelength-dispersive X-ray spectroscopy (WDS) were also performed.

**Figure 7:**
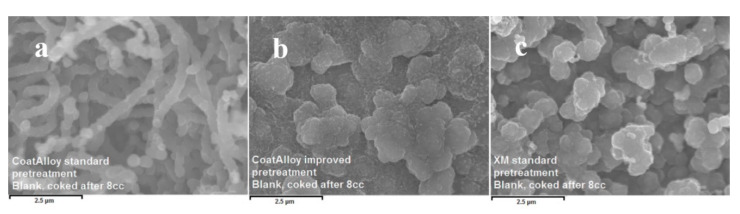
After 8 cracking cycles (cc): coked CoatAlloy™ after the standard treatment (**a**), coked CoatAlloy™ after the steam/air only treatment (**b**) and coked 25/35 Cr/Ni after a standard treatment (**c**). Magnification: 15000 ×, Accelerating Voltage: 10 kV.

In Figure 7 the effect of the steam/air pretreatment (Figure 7b) on the coke structure is visible. A more porous and finer coke structure can be observed on the CoatAlloy^™^ pretreated with the standard pretreatment for Fe-Ni-Cr Alloys (Figure 7a) [7]. When no N_2_, but only steam with air is fed during the pretreatment and decoking phases, the structures noted are more similar with the reference material (Figure 7c), certainly less porous. The difference can be linked with the lower amount of available surface and therefore less active sites promoting coke formation, justifying the improved performance of CoatAlloy™ after pure steam treatment.

**Figure 8:**
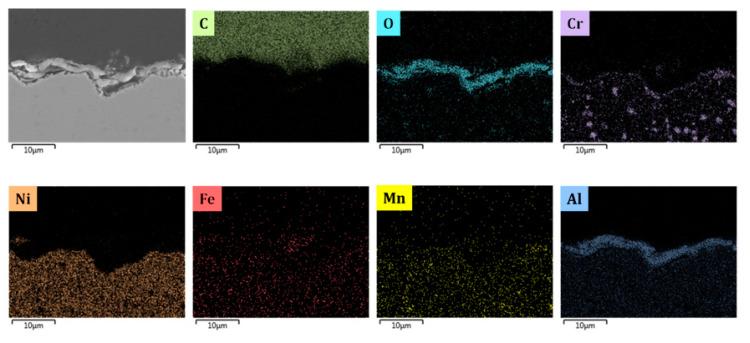
Cross-sectional elemental mapping of a CoatAlloy™ sample subjected to 14 h nitration. Magnification: 3000 ×, accelerating voltage: 15 kV.

**Figure 9:**
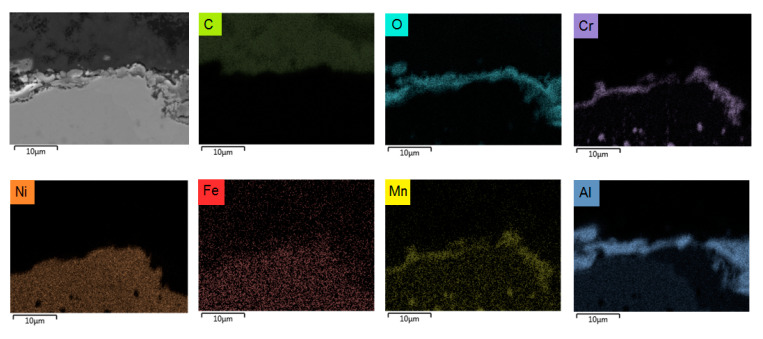
Cross-sectional elemental mappings of a CoatAlloy™ sample after application of the standard pretreatment. Magnification: 3000 ×, accelerating voltage: 15 kV.

**Figure 10:**
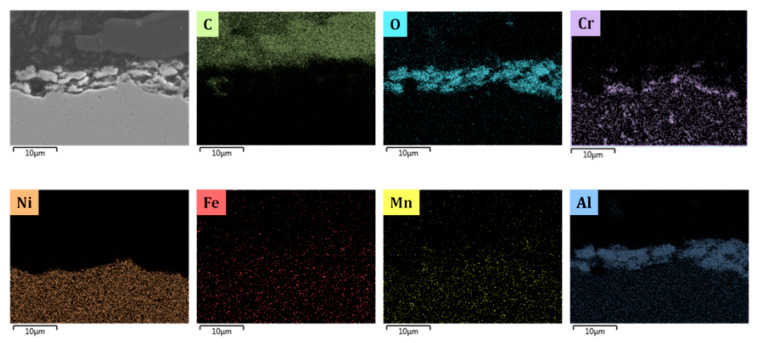
Cross-sectional elemental mappings of a CoatAlloy™ sample subjected to the steam/air pretreatment Magnification: 3000 ×, accelerating voltage: 15 kV.

**Figure 11:**
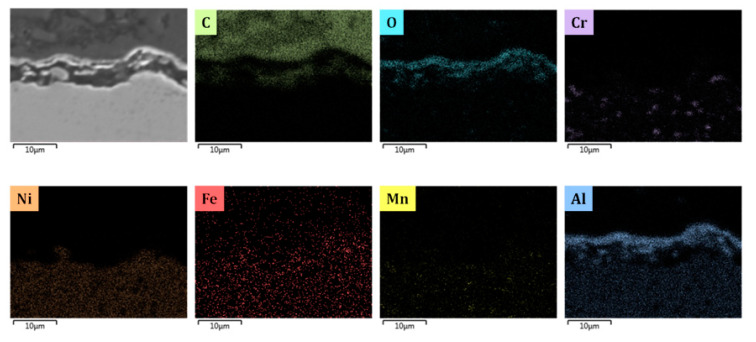
Cross-sectional elemental mapping of a CoatAlloy™ sample subjected to the steam/air pretreatment followed by presulfiding. Magnification: 3000 ×, accelerating voltage: 15 kV.

Figure 5, Figure 8, Figure 9, Figure 10 and Figure 11 show the cross-sectional analyses of the CoatAlloy™ under the different applied conditions. The untreated coating consists out Al and O, having a thickness of 5–8 μm (see Figure 5). In Figure 8, a sample exposed to N_2_ for 14 h at 1023 K is shown. A uniform layer of Cr beneath the alumina coating layer can be observed, together with a decreased thickness of the coating layer. The latter is also more heterogeneous. The same Cr layer can be seen also in Figure 9, here overlaying with Mn and O, implying the formation of a Cr and Mn oxide layer under the coating.

When N_2_ is excluded from the pretreatment, a thin layer of Cr is again observed under the coating, however the oxygen is not present, implying that penetration of the O to the layers under the coating was not possible without the N_2_ presence during the pretreatment. The thickness of the coating remains the same as were observed in the fresh, untreated coating.

By comparing Figure 9 and Figure 10, it can be observed that the steam/air pretreatment keeps the coating thickness unaffected, leaving the Cr layer isolated underneath the alumina layer. This can support the idea of a more stable α alumina layer for the steam/air treatment in comparison with a mix of alumina γ and α layer formed after application of the optimal pretreatment for Fe-Ni-Cr alloys [7].

Similarly, Figure 11 depicts the coating after the steam/air pretreatment combined with a presulfiding step. No significant effect is noted, while the thin Cr layer beneath the coating is absent and the thickness of the alumina layer unaffected, proving good resistance of the coating towards inner oxidation.

**Figure 12:**
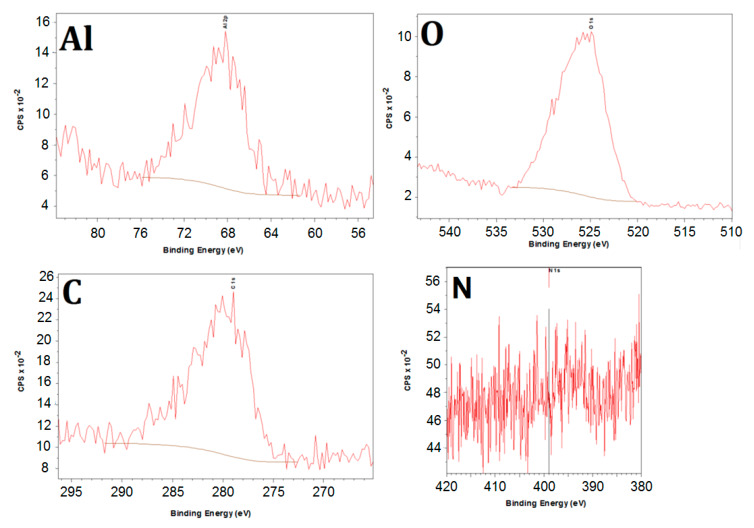
XPS integrated spectra for C, O, N and Al for the CoatAlloy™ sample after the typical pretreatment for Fe-Ni-Cr alloys.

To further evaluate the effect of N_2_ on the surface of the coating, additional XPS (Figure 12) analyses are performed. The results show that no nitrogen could be detected on the surface. For the conducted analysis the detection limit is 0.1 to 1 at%, so if nitrogen is present on the surface it should be in relatively low concentrations or it simply reacts with the surface oxides by removing active oxygen atoms from the surface. The latter was below the detection limit of the analytical section of the used set-up.

**Figure 13:**
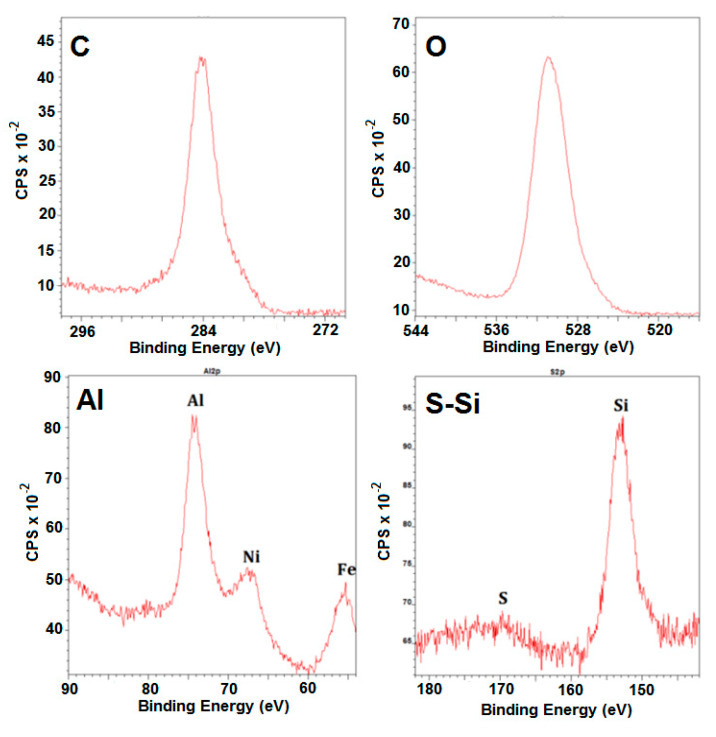
XPS-spectra for the different elements in sulfided coated sample.

The same conclusion can be drawn from the XPS analysis (Figure 13) of the presulfided coated samples. It has been found in similar work that sulfur influences the physical and chemical nature of catalytically active sites [52]. Sulfur stimulates a process of carbon-induced corrosion, which particularly can deteriorate chromia-forming alloys, whereas alumina scales are resistant. In our work, insignificant amounts of sulfur are identified, however the assumption of the sulfides’ formation on the surface of the coating is not valid.

As a last step, an analysis with WDS is carried out, which allows penetrating deeper into the sample at detection limits of 0.01 wt% to detect nitrogen and sulfur. In all the measurements either no weight percentage is obtained or the error margin is larger than the peak of nitrogen itself, because of peak overlap. Nevertheless the lack of a larger nitrogen peak already suggests that there is likely an insignificant amount of nitrogen on the surface of the samples. The WDS measurements therefore indicate that no nitrites are formed on the surface of the sample after prenitration, in accordance with the observations of the XPS analyses.

WDS analyses for sulfur are a bit better, giving numerical observations, as summarized in Table 5 and Table 6. However, again judging by these tables, no link can be made between the Sulfur identified and the pretreatment conditions of the samples.

## 4. Conclusions

In this work, for the first time the coking tendency of a CoatAlloy™ coating has been quantitatively evaluated and compared to a classical high temperature 25/35 Cr/Ni alloy based on the on-line measured coking rates over multiple coking/decoking cycles. It has been experimentally proven that the use of that coating has industrial potential due to the mitigation of coking in comparison with the reference material if mainly steam and air is used for its pretreatment.

Overall, the addition of sulfur on-stream or as pretreatment leads to a decrease in carbon oxides formation. Apart from the blank experiments, no significant observable differences in product yields were noted between the reference material and the passivating formulation under the different conditions.

The coating generated almost two times more CO compared to the reference material for the blank runs. An additional presulfiding step before cracking, when no sulfur was added during cracking, led to a 20% decrease of carbon oxides formation for both the reference and the coated sample. The reduction was more pronounced for the blank run; a factor of five and two mitigation of CO and CO_2_, respectively, was observed for the reference, 25/35 Cr/Ni, while the diminution for CoatAlloy was eight and two, for CO and CO_2_, respectively.

A treatment with nitrogen should be avoided as this results in a coking rate for CoatAlloy™ that is two to five times more than the 25/35 Cr/Ni material. Also presulfiding is not advised for CoatAlloy™ because it results in an average increase of 20% in the asymptotic coking rate for CoatAlloy™ compared to the reference material. When DMDS is continuously added initially 25/35 Cr/Ni performs almost two times better than CoatAlloy™. However, after aging the performance of the barrier coating improves significantly, while the reference material seems to remain rather stable. In that way, applying the coating results in 10 and 50% lower coking rates for the catalytic and pyrolytic rate, respectively, in comparison with the reference material, showing a very good potential for industrial use. Overall the coating seems to remain stable after application of presulfiding, cyclic aging and continuous addition of DMDS, which paves the way to industrial implementation.

## Figures and Tables

**Table 1:** Composition of the reference alloy [47].

C	Mn	Si	Ni	Cr	Mo	Nb	Cu	Fe	Ti, Zr, W
0.45–0.5	<1.5	1.5–2	33–36	24–27	<0.5	0.5–1	<0.25	>31.18	add

**Table 2:** Schematic overview of the coking-decoking experiments with cyclic aging.

Process Step		Alloy	Duration	Temperature (K)	Gas Feed Flow (Nl/s)			Water Flow (10^−6^ kg s^−1^)
					N_2_	Ethane	Air	
**Initial pre-oxidation**		**Fe-Ni-Cr & CoatAlloy**	12–14 h	1023	—	—	0.0067	—
**Pretreatment**	**Pretreatment (or Decoking)**	**Fe-Ni-Cr**	30 min	from 1023 to 1173 K	0.0083	—	0.0083	—
		**CoatAlloy**			—	—	0.0083	6.7
	**Steam Treatment**	**Fe-Ni-Cr & CoatAlloy**	15 min	1173 K	—	—	0.0083	6.7
PreS *	**Presulfiding**	**Fe-Ni-Cr & CoatAlloy**	30 min	1100 K	—	—	—	5.6
**Cracking**	**1st cracking cycle**	**Fe-Ni-Cr & CoatAlloy**	6 h	1173 K	—	0.0241	—	9.7
**Decoking**	**Pretreatment (or Decoking)**	**Fe-Ni-Cr**	30 min	from 1023 to 1173 K	0.0083	—	0.0083	—
		**CoatAlloy**			—	—	0.0083	6.7
	**Steam Treatment**	**Fe-Ni-Cr & CoatAlloy**	15 min	1173 K	—	—	0.0083	6.7
PreS *	**Presulfiding**	**Fe-Ni-Cr & CoatAlloy**	30 min	1100 K	—	—	—	5.6
**Cracking**	**next cracking cycle**	**Fe-Ni-Cr & CoatAlloy**	6 h	1173 K	—	0.0241	—	9.7

* PreS: presulfiding is applied after the pretreatment only for the presulfided experiments.

**Table 3:** Location of the XPS characteristic peaks for the most relevant elements for the studied materials.

Element + Orbital	XPS Characteristic Peak [eV]
Si_2p_ + Al_2s_	95–130
S_2p_	155–175
C_1s_	275–295
N_1s_	390–410
O_1s_	520–540
Cr_2p_	565–605
Mn_2p_	635–660
Fe_2p_	700–740
Ni_2p_	845–900

**Table 4:** Summary of the coking experiments and main results.

Material	25/35 Cr/Ni	CoatAlloy	CoatAlloy	25/35 Cr/Ni	CoatAlloy	25/35 Cr/Ni	CoatAlloy	25/35 Cr/Ni	CoatAlloy
	Standard	Standard	Steam/Air	Standard	Steam/Air	Standard	Steam/Air	Standard	Steam/Air
Pretreatment id	Blank			PreS		CA		CA+PreS	
**Cracking Temperature (K)**	1173			1173		1173		1173	
**N_2_ during stabilization**	no			no		no		no	
**dilution**	0.33			0.33		0.33		0.33	
**CA DMDS (ppmw S per HC)**	0			0		41		41	
**PreS (ppmw DMDS per H_2_O)**	0			500		0		500	
**cc**	Coke formed (mg) *								
**1**	1.29	2.01	1.61	2.46	5.60	7.55	14.28	19.15	36.16
**2**	1.26	2.08	1.65	2.18	3.83	8.38	16.89	12.24	36.04
**3**	1.41	2.47	1.93	2.52	4.71	8.66	11.52	12.84	37.09
**4**	0.60	1.26	0.96	0.64	1.77	4.12	2.39	3.73	4.68
**5**	0.67	1.97	1.50	1.19	1.66	4.55	2.34	4.08	4.52
**6**	0.69	1.18	0.94	1.65	1.88	4.53	2.54	4.54	4.47
**7**	0.47	1.17	0.91	1.91	1.51	4.51	2.44	2.91	4.73
**8**	1.19	5.08	4.13	2.84	4.97	11.46	9.69	11.21	16.42
**cc**	**Initial coking rate [10^−6^ kg/s/m^2^]**								
**1**	0.71	0.72	0.48	0.63	1.52	3.03	5.15	6.28	8.79
**2**	0.67	0.73	0.49	0.87	1.30	4.74	7.07	3.39	8.72
**3**	0.86	0.83	0.55	1.04	1.86	4.23	3.26	3.63	8.80
**4**	0.85	1.56	1.04	0.91	1.95	5.85	2.69	5.28	4.93
**5**	0.96	2.44	1.63	1.68	1.82	6.45	2.63	5.78	4.50
**6**	0.97	1.46	0.98	2.34	2.31	6.42	2.82	6.43	4.70
**7**	0.67	1.44	0.96	2.71	1.87	6.39	2.71	4.12	4.86
**8**	0.63	2.56	1.71	1.36	1.77	5.29	4.40	5.42	5.03
**cc**	**Asymptotic coking rate [10^−6^ kg/s/m^2^]**								
**1**	0.22	0.35	0.24	0.57	0.87	1.53	2.14	4.17	6.85
**2**	0.22	0.37	0.24	0.45	0.55	1.43	2.04	2.79	6.86
**3**	0.23	0.44	0.30	0.51	0.61	1.61	1.85	2.91	7.14
**8**	0.21	0.74	0.50	0.53	0.73	2.17	1.01	2.09	2.67
**Products**	**Averaged Yields (wt %)**								
**H_2_**	4.24	4.26	4.23	4.26	4.23	4.25	4.23	4.26	4.24
**CO_2_**	0.006	0.007	0.006	0.004	0.004	0.003	0.003	0.002	0.003
**CO**	0.05	0.11	0.09	0.04	0.08	0.01	0.01	0.01	0.01
**C_2_H_6_**	30.13	29.88	29.94	29.84	29.86	30.21	29.96	30.02	29.88
**C_2_H_4_**	49.86	50.18	50.31	50.06	50.39	49.76	50.42	49.87	50.41
**C_3_H_6_**	0.74	0.76	0.75	0.76	0.77	0.76	0.77	0.76	0.77

* In all coking results a 10% error should be accounted.

**Table 5:** Sulfur detection in wt% for the three Fe-Ni-Cr samples: Fe-Ni-Cr sample without sulfur addition, presulfided and pretreated before cracking without presulfiding. The error margin is 0.001 wt% for all measurements. Acceleration voltage: 15 kV.

Sample	Position 1	Position 2	Position 3	Position 4	Average
Fresh Fe-Ni-Cr	0.014	0.015	0.029	0.035	0.02325
PreS Fe-Ni-Cr	0.019	0.024	0.013	0.015	0.01775
Pretreated Fe-Ni-Cr	0.006	0.003	0.004	0.004	0.00425

**Table 6:** Sulfur detection in wt% for the three CoatAlloy™ samples: CoatAlloy™ sample without sulfur addition, presulfided and pretreated before cracking without presulfiding. The error margin is 0.001 wt% for all measurements. Acceleration voltage: 15 kV.

Sample	Position 1	Position 2	Position 3	Position 4	Average
Fresh CoatAlloy	0.012	0.034	0.025	0.017	0.02200
PreS CoatAlloy	0.024	0.014	0.018	0.017	0.01825
Pretreated CoatAlloy	0.016	0.023	0.011	0.022	0.01800

## Supplementary Materials

The following information is available online at https://www.mdpi.com/1996-1944/13/9/2025/s1. Figure S1: Processing sequence for the coking rates determination, Figure S2: Cross-section build-up of the CoatAlloy™ passivating coating.

## Nomenclature

A	Atomic mass of an element [g·mol^−1^]
Blank	The cracking experiment in which no DMDS is added during cracking or the pretreatment
CC	cracking cycle
CA	The experiment that DMDS diluted in steam is continuously added during cracking.
CA+PreS	The experiment in which continuous addition of DMDS is combined with presulfiding.
Coking	The process during cracking that coke is depositing on the sample
Cyclic aging	A cyclic process that simulates the real behavior of an industrial cracker using the repetition of coking and decoking of the material
Decoking	The process after cracking that the coke is burned into carbon oxides using steam or air or mixture thereof
DMDS	Dimethyl disulfide
FID	Flame ionization detector
F	Flow rate [10^−6^ kg·s^−1^]
H	Penetration depth [µm]
HTA	High temperature alloy
JSR	Jet-stirred reactor
PreS	Presulfiding experiment. The experiment in which presulfiding is applied before cracking
r_c_	coking rate [kg·s^−1^·m^−2^]
r_c, initial_	initial catalytic coking rate [kg·s^−1^·m^−2^]
r_c, asymptotic_	Asymptotic/pyrolytic coking rate [kg·s^−1^·m^−2^ ]
r_x-y_	Average coking rate in between the given time intervals [kg·s^−1^·m^−2^]
RGA	Refinery gas analyzer
S	Surface area of the sample [m^2^]
sample	The metal sample cut from real industrial coil that is placed in the JSR
SEM	Scanning electron microscopy
TCD	Thermal conductivity detector
T	Temperature [K]
V	Accelerating voltage [kV]
WDS	Wavelength-dispersive spectroscopy
XPS	X-ray photoelectron spectroscopy
z	Atomic number
δ	Dilution [kg·kg^−1^]
ρ	Density of the material [g·cm^−3^]
τ	Mean residence time [s]
