# Supplementary material for: Alumina-based Coating for Coke Reduction in Steam Crackers
Source: Materials (Basel). 2020 Apr 26;13(9):2025. doi: 10.3390/ma13092025 (PMC7254218; doi:10.3390/ma13092025)
Supplement: Supplementary file 1 [file materials-13-02025-s001.pdf]

materials

Supplementary Materials

Alumina-based Coating for Coke Reduction in Steam Crackers

Stamatis A. Sarris, Steffen H. Symoens, Natalia Olahova, Marie-Françoise Reyniers, Guy B. Marin and Kevin M. Van Geem *

Department of Materials, Textiles and Chemical Engineering, University of Gent, Technologiepark 914, 9052 Gent, Belgium; stamatisasarris@gmail.com (S.A.S.); steffen.symoens@ugent.be (S.H.S.); natalia.olahova@kubota.com (N.O.); MarieFrancoise.Reyniers@UGent.be (M.-F.R.); guy.marin@ugent.be (G.B.M.)

* Correspondence: Kevin.VanGeem@ugent.be

Coke Formation Mechanisms

The carbon deposition process during thermal cracking in the presence, or not, of steam, is a quite complex phenomenon. It has been extensively described in literature [1–5] that it mainly consists of three mechanisms; catalytic coke formation, pyrolytic coke growth from existing carbon layers onwards and gas phase coking.

The initial phase of carbon deposition is the formation of a porous network of carbon filaments, catalyzed typically by Ni and Fe present at the surface of the reactor walls. It is widely accepted that catalytic carbon formation [6–10] on metallic surfaces involves surface reactions, diffusion and precipitation of carbon. As a first step, a hydrocarbon molecule is chemisorbed on a metal crystallite of the surface. Dehydrogenation of the R-CH groups takes place with the hydrogen atoms recombining and desorb into the gas phase. As a result, the carbon atoms are formed at the surface, dissolve in and diffuse through the metal particle. The carbon accumulation in the particle causes a pressure build up at the dislocations and the grain boundaries, which may exceed the tensile strength of the metal.

Potentially, the metal particle is then lifted from the surface and carbon crystallizes at the rear end of the particle. Growing stems are developed that carry crystallites on their top. The precipitation of the carbon can give rise to structural deficiencies in the carbon lattice, thereby creating reactive carbon sites along this layer. Additional hydrocarbon molecules from the gas phase are incorporated at these sites causing addable filamentous coke growth. In that way, a porous layer of interwoven filaments is developed. The metal particles at the top of the whiskers are becoming more accessible from the gas phase, therefore the deposition rate increases, while the diffusion rate through the metal remains stable. Carbon migration over the metal surface occurs, surrounding the carbon stems. At this point, surface carbon can occur, encapsulating the metal and terminating any further coke growth.

Dissociative chemisorption of water molecules on the metal particles produces highly reactive oxygen atoms that react with surface carbon to from carbon monoxide, which desorbs in the gas phase. This reaction prevents the fast encapsulation of the metal tip of the filament and therefore the termination of this reaction, in other words the “deactivation of the metallic active site”. How quickly these phenomena are occurring is a matter of relative kinetics of carbon growth, gasification, dissolution and diffusion. Certainly, the properties of the alloy are very important in all the mechanisms, however in this study the composition was kept stable to isolate the effect of roughness.

The heterogeneous non-catalytic mechanism, also known as pyrolytic coking, is the major source of coke in an industrial cracker, since it takes place over the complete run length. The coke in contact with the gas phase looks like a succession of several discrete layers, deriving from the coke-gas interface [11,12]. Coke formation is described by the reaction of gas phase precursors with active centers of the surface. Kopinke et al [1] have provided a general scale for the coking tendency of

several precursors. For example, acetylene, anthracenes, cyclic naphthenes and aromatics possess a high tendency for coke formation. Ethylene is not the most reactive one, however due to its high concentration is very important. No significant differences of the relative constants of a particular hydrocarbon were found for different materials [1], supporting that the active centers are radical in nature and positioned in the coke matrix. The radicals can be generated by hydrogen abstraction from the partially dehydrogenated carbon layer being determined by the gas phase composition and the available coked reactor surface [13]. The coke radicals react by addition with unsaturated molecules and radicals from the gas phase. After dehydrogenation, graphitic layers are formed [14].

The last mechanism, also known as homogeneous non-catalytic coking results from a sequence of molecular and/or radical reactions in the gas phase. These lead to high molecular weight polynuclear aromatic compounds which remain solid even at high temperatures. As a result, they collide at the wall and integrate in the coke layer [15]. In this work, the latter mechanism contributes very little, since there are not a lot heavy compounds generated in the gas phase due the light feedstock (ethane).

Coking Rates and Coking Curve

The coke deposition on each sample is measured over time by continuously weighing the mass of the sample. This allows the determination of the total amount of coke after every cracking cycle, as well as the calculation of the initial catalytic coking rate and the asymptotic pyrolytic coking rate. Similarly with previous work [16], the coking rate is determined as:

$$R_c = \frac{m_{t_2} - m_{t_1}}{t_2 - t_1}\frac{1}{S} \quad (1)$$

where R_c is the coking rate in $kg \cdot s^{-1} \cdot m^{-2}$, mt_j the mass of coke at time j in kg, t_j the experimental time at instant j in s and S the surface area of the coupon in m^2.

In this work, the mean value of the coking rate between 15 min and 60 min is defined as the initial or catalytic coking rate, i.e. characterizing the catalytic coking behavior of the sample. The asymptotic or pyrolytic coking rate is related to the pyrolytic coking mechanism and is reported as the mean measured coking rate between the 5th and 6th hour of cracking (see Figure 3).

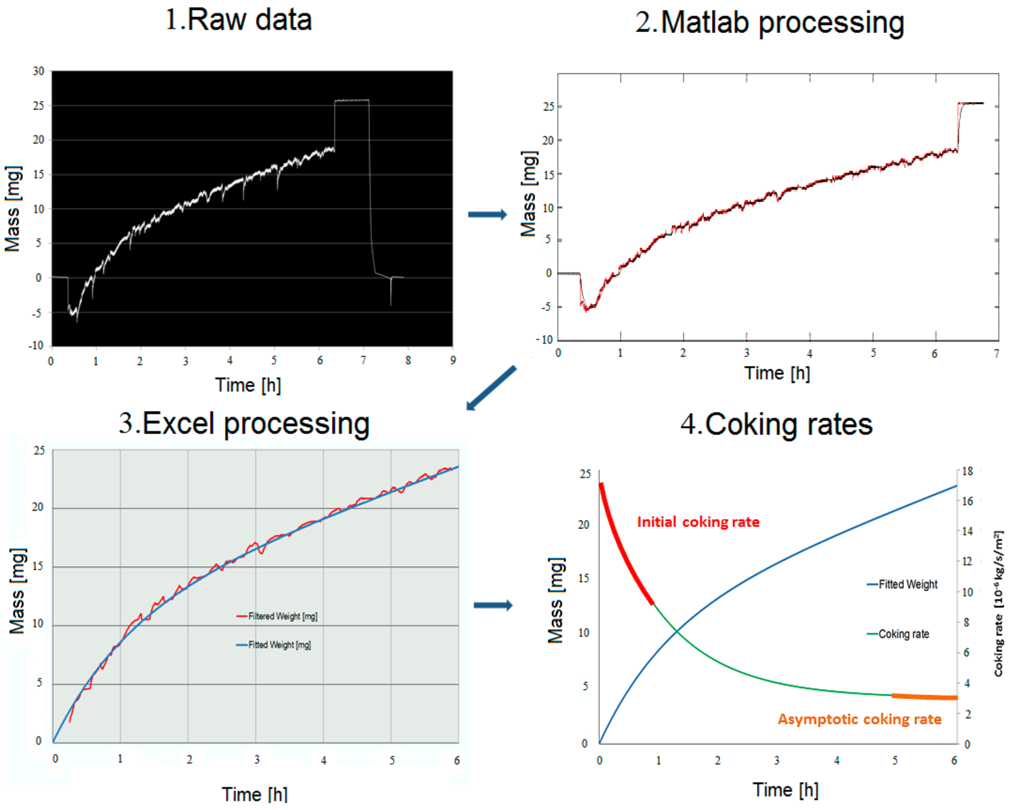

Figure S1. Processing sequence for the coking rate determination**.**

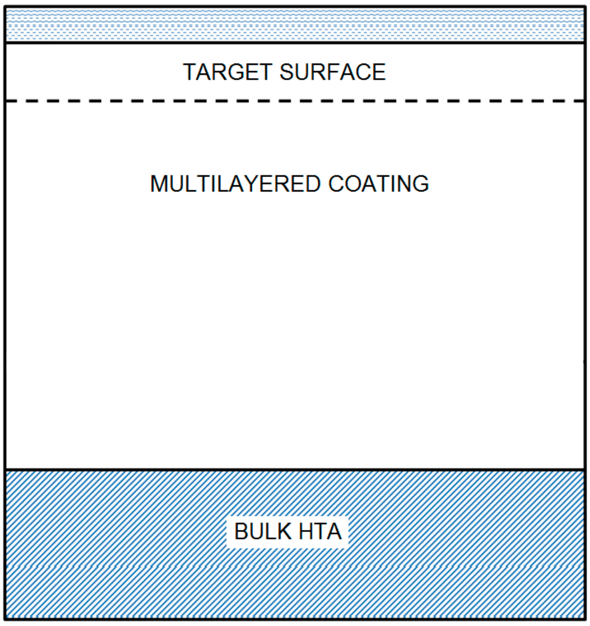

Figure S2. Cross section build-up of the CoatAlloy™ passivating coating [16]**.**

References

1. Kopinke, F.D.; Zimmermann, G.; Nowak, S. On the mechanism of coke formation in steam cracking—conclusions from results obtained by tracer experiments. *Carbon* **1988**, *26*, 117–124, doi:10.1016/0008-6223(88)90027-9.
2. Cai, H.; Krzywicki, A.; Oballa, M.C. Coke formation in steam crackers for ethylene production. *Chem. Eng. Process. Process Intensif.* **2002**, *41*, 199–214, doi:10.1016/S0255-2701(01)00135-0.
3. Albright, L.F.; Marek, J.C. Mechanistic model for formation of coke in pyrolysis units producing ethylene. *Ind. Eng. Chem. Res.* **1988**, *27*, 755–759, doi:10.1021/ie00077a006.
4. Lahaye, J.; Badie, P.; Ducret, J. Mechanism of carbon formation during steamcracking of hydrocarbons. *Carbon* **1977**, *15*, 87–93, doi:10.1016/0008-6223(77)90022-7.
5. Wauters, S.; Marin, G.B. Kinetic Modeling of Coke Formation during Steam Cracking. *Ind. Eng. Chem. Res.* **2002**, *41*, 2379–2391, doi:10.1021/ie010822k.
6. Baker, R.T.K.; Yates, D.J.C.; Dumesic, J.A. Filamentous Carbon Formation over Iron Surfaces. In *Coke Formation on Metal Surfaces*; AMERICAN CHEMICAL SOCIETY: Washington, DC, USA, 1983; Volume 202; pp. 1–21.
7. Lyle, F.A.; Baker, R.T.K. *Coke Formation on Metal Surfaces*; AMERICAN CHEMICAL SOCIETY: Washington, DC, USA, 1983; Volume 202; pp. 332.
8. Figueiredo, J.L. Reactivity of coke deposited on metal surfaces. *Mater. Corros.* **1999**, *50*, 696–699, doi:10.1002/(SICI)1521-4176(199912)50:12<696::AID-MACO696>3.0.CO;2-I.
9. Snoeck, J.-W.; Froment, G.; Fowles, M. Filamentous carbon formation and gasification: Thermodynamics, driving force, nucleation, and steady-state growth. *J. Catal.* **1997**, *169*, 240–249.
10. Bonnet, F.; Ropital, F.; Berthier, Y.; Marcus, P. Filamentous carbon formation caused by catalytic metal particles from iron oxide. *Mater. Corros.* **2003**, *54*, 870–880.
11. Ranzi, E.; Dente, M.; Goldaniga, A.; Bozzano, G.; Faravelli, T. Lumping procedures in detailed kinetic modeling of gasification, pyrolysis, partial oxidation and combustion of hydrocarbon mixtures. *Prog. Energy Combust. Sci.* **2001**, *27*, 99–139.
12. Kopinke, F.D.; Zimmermann, G.; Reyniers, G.C.; Froment, G.F. Relative rates of coke formation from hydrocarbons in steam cracking of naphtha. 2. Paraffins, naphthenes, mono-, di-, and cycloolefins, and acetylenes. *Ind. Eng. Chem. Res.* **1993**, *32*, 56–61, doi:10.1021/ie00013a009.
13. Wauters, S.; Marin, G.B. Computer generation of a network of elementary steps for coke formation during the thermal cracking of hydrocarbons. *Chem. Eng. J.* **2001**, *82*, 267–279, doi:10.1016/S1385-8947(00)00354-5.

14. Reyniers, G.C.; Froment, G.F.; Kopinke, F.-D.; Zimmermann, G. Coke Formation in the Thermal Cracking of Hydrocarbons. 4. Modeling of Coke Formation in Naphtha Cracking. *Ind. Eng. Chem. Res.* **1994**, *33*, 2584–2590, doi:10.1021/ie00035a009.
15. Bach, G.; Zimmermann, G.; Kopinke, F.-D.; Barendregt, S.; van den Oosterkamp, P.; Woerde, H. Transfer-Line Heat Exchanger Fouling during Pyrolysis of Hydrocarbons. 1. Deposits from Dry Cracked Gases. *Ind. Eng. Chem. Res.* **1995**, *34*, 1132–1139, doi:10.1021/ie00043a015.
16. Paradigm. *Petrochemical: CoatAlloy™*; Paradigm Shift Technologies Inc.: Toronto, ON, Canada, 2015.

© 2020 by the authors. Licensee MDPI, Basel, Switzerland. This article is an open access article distributed under the terms and conditions of the Creative Commons Attribution (CC BY) license (http://creativecommons.org/licenses/by/4.0/).
